# Addressing Sleep Disorders in Psychiatry: Comparing the Use of Melatonin, Trazodone, and Doxepin

**DOI:** 10.7759/cureus.76507

**Published:** 2024-12-28

**Authors:** Beena Mamoon, Amber Nawaz, Muhammad Iftikhar Khattak, Fehmida Amir, Amna Akbar, Tashbiha E Batool, Shahid Khan

**Affiliations:** 1 Department of Psychiatry, Kulsum International Hospital, Islamabad, PAK; 2 Department of Psychiatry, Azad Jammu and Kashmir Medical College, Muzaffarabad, PAK; 3 Department of Research and Development, Health Services Academy, Islamabad, PAK; 4 Department of Medicine, Sarosh Hospital Diagnostic Center, Muzaffarabad, PAK; 5 Department of Emergency and Accident, District Headquarter Hospital, Jhelum Valley, Hattian Bala, PAK; 6 Department of Family Medicine, Holy Family Hospital, Rawalpindi, PAK

**Keywords:** clinical global impression-improvement, doxepin, efficacy, melatonin, pittsburgh sleep quality index, psychiatric patients, sleep disorders, tolerability, trazodone

## Abstract

Introduction

Sleep disorders are prevalent among psychiatric patients, and pharmacological treatments such as melatonin, trazodone, and doxepin are commonly prescribed. This study aimed to assess the efficacy and acceptability of these three medications in improving sleep quality and reducing daytime drowsiness in psychiatric patients.

Methodology

A total of 175 psychiatric patients with sleep disturbances participated in this cohort study at the Abbas Institute of Medical Sciences, Muzaffarabad, Pakistan.Participants were initially randomized, with assignments subsequently reviewed and confirmed by physicians based on clinical considerations, into one of three therapy groups: doxepin, trazodone, or melatonin. They were monitored over the course of six months, from February to July 2024. The Pittsburgh Sleep Quality Index (PSQI) was used to measure sleep quality, the Epworth Drowsiness Scale (ESS) was used to measure daytime drowsiness, and the Clinical Global Impression-Improvement (CGI-I) scale was used to determine clinical improvement. Pre- and post-treatment data were analyzed in IBM SPSS Statistics for Windows, Version 26.0 (Released 2019; IBM Corp., Armonk, New York, United States) using statistical techniques such as paired t-tests, ANOVA, and chi-square tests.

Results

Trazodone, doxepin, and melatonin were evaluated for their effectiveness and tolerability in improving sleep quality and reducing daytime drowsiness among 175 psychiatric patients (n=58 for melatonin, n=59 for trazodone, n=58 for doxepin). Trazodone showed the greatest improvement in sleep quality, with significant reductions in PSQI scores at six months (mean decrease = 7.0, SD = 1.9) and the highest CGI-I improvement rates (n=59, 76%, p = 0.02), but it was associated with frequent adverse effects, including morning grogginess (n=59, 15%, p = 0.03) and orthostatic hypotension (n=59, 10%, p = 0.02). Doxepin significantly enhanced sleep continuity (PSQI reduction = 6.8, SD = 2.1) and had a better tolerability profile than trazodone but was linked to dry mouth (n=58, 13%, p = 0.04). Melatonin, while slightly less effective in improving sleep quality (PSQI reduction = 6.1, SD = 2.0), had the fewest adverse effects, including the lowest rates of morning grogginess (n=58, 5%, p = 0.03) and dizziness (n=58, 10%, p = 0.41), and significantly reduced daytime drowsiness (ESS decrease = 3.9, SD = 1.7, p = 0.04). These findings highlight trazodone and doxepin as the most effective treatments, while melatonin offers better tolerability for patients concerned about adverse effects.

Conclusion

In psychiatric patients, trazodone was the most successful medication for enhancing sleep quality; however, other groups cannot use it due to its adverse effects. For patients who were more likely to have side effects, melatonin was a safer option, but doxepin offered a good balance between effectiveness and tolerability.

## Introduction

People with psychiatric conditions frequently suffer from sleep disturbances, which greatly increase the difficulty of therapy and negatively impact patient outcomes [1]. Sleep problems can complicate patient care by exacerbating the symptoms of mental health disorders and impairing the efficacy of psychiatric therapies [2]. Sleep disturbances are a common feature of mental health disorders such as schizophrenia, bipolar disorder, generalized anxiety disorder, and major depressive disorder [3]. Insomnia, hypersomnia, or disturbed sleep architecture are common symptoms of these diseases, which call for specialized therapy techniques to enhance sleep quality and, in turn, mental health in general [4]. Because mental health symptoms, drug side effects, and the patient's overall medical profile interact, treating sleep disturbances in people with psychiatric conditions is challenging. Even if non-pharmacological treatments, including cognitive-behavioral therapy for insomnia (CBT-I), are advised and successful, not all patients may be able to use them because of issues with accessibility, adherence, and the urgent need for sleep relief in extreme situations [5]. In order to address sleep disruptions in psychiatric patients, pharmaceutical therapies continue to be a standard practice.

Melatonin, trazodone, and doxepin are three different pharmacological therapies with different profiles and mechanisms among the alternatives available. The pineal gland naturally produces the hormone melatonin, which controls circadian rhythms and is a popular treatment for insomnia based on the circadian cycle [6]. Melatonin is frequently chosen because of its non-habit-forming nature and generally benign side-effect profile [7]. It is a readily available and typically well-tolerated over-the-counter supplement, which makes it a desirable choice, especially for patients whose sleeplessness may be caused by abnormalities in their circadian rhythm [8]. Melatonin's efficacy in treating insomnia in psychiatric populations is still not entirely obvious, though, as different studies have produced differing findings, particularly when mental health issues interfere with sleep patterns [9]. Because of its strong calming effects at low dosages, trazodone, which was first created as an antidepressant, is now frequently taken off-label for insomnia. It helps people with mood disorders initiate and maintain sleep through its mechanism of action as a serotonin receptor antagonist and reuptake inhibitor [10].

Trazodone has a comparatively low risk of dependence compared to many conventional hypnotics; yet, it can cause side effects such as priapism, orthostatic hypotension, and morning grogginess [11]. Even if these adverse effects are tolerable, they should be taken into account, especially for older individuals or those who already have cardiovascular disease. Because of its potent antihistaminic qualities, doxepin, a tricyclic antidepressant, is very useful in maintaining sleep and is authorized at low dosages for the treatment of insomnia. Low-dose doxepin is generally well-tolerated, seen as safe for long-term usage, and has a low risk of reliance, in contrast to greater doses used in the treatment of depression [12]. However, in some patient groups, its sedative effects may be followed by adverse effects such as weight gain, dry mouth, and anticholinergic effects, which call for caution [13]. Despite these limitations, doxepin is a useful treatment choice because of its ability to keep patients with psychiatric problems asleep and lessen nightly awakenings [14].

Current research often overlooks comprehensive evaluations within larger psychiatric populations, focusing instead on these medications in the general population or specific psychiatric cohorts. Moreover, many studies lack head-to-head comparisons, limiting the valuable insights clinicians can gain regarding the side effect profiles and relative efficacy of these treatments. The objective of this study is to evaluate and compare the efficacy, safety, and tolerability of melatonin, trazodone, and doxepin in treating sleep disturbances in psychiatric patients. By conducting a direct head-to-head comparison, this study aims to provide comprehensive data on how these medications impact sleep quality and reduce daytime drowsiness and their side effect profiles, and thus, address a significant gap in the existing literature. Ultimately, the goal is to offer valuable insights that will help refine pharmacological approaches to improve patient outcomes and enhance the quality of sleep in individuals with psychiatric disorders.

## Materials and methods

Study design and setting

This cohort study was conducted at the Abbas Institute of Medical Sciences, Muzaffarabad, Azad Kashmir, Pakistan. The study was approved by the Abbas Institute of Medical Sciences (approval number: 7828/AIMS/2024). A total of 175 patients with associated sleep problems and psychiatric disorders were enrolled. Melatonin, trazodone, and doxepin are three therapeutic drugs that were evaluated and compared for their effectiveness, safety, and tolerability in treating sleep disturbances in this group. In order to evaluate therapy outcomes, each participant was monitored for six months from February to July 2024.

Sample size calculation

A power analysis based on prior research assessing pharmaceutical treatment for sleep disorders was used to select the sample size of 175 [15]. With a significance threshold of 0.05, a minimum of 80% power was targeted to identify a clinically significant difference in sleep quality between treatment groups. A total of 175 participants was judged sufficient to guarantee solid and trustworthy results across the three treatment arms, assuming a 10% dropout rate.

Participant selection

Participants were chosen from the inpatient and outpatient psychiatric units of the Abbas Institute of Medical Sciences. Patients with a diagnosed psychiatric disease, such as major depressive disorder, generalized anxiety disorder, or bipolar disorder, with clinically severe sleep problems as determined by the Pittsburgh Sleep Quality Index (PSQI), were eligible to participate. Patients had to be between the ages of 18 and 65 years. A history of substance misuse, current use of other sedative or hypnotic medications, serious medical conditions, or contraindications to the research medications were among the exclusion criteria.

Group allocation and treatment protocols

Participants were initially randomized into groups, with assignments subsequently reviewed and confirmed by physicians based on clinical considerations. Melatonin (3 mg daily at bedtime), trazodone (50 mg daily at bedtime), and doxepin (10 mg daily at bedtime) were the treatments administered to the groups respectively. Every night, participants were told to take their prescribed medication and to report any side effects or changes in the quality of their sleep. In order to make sure they were following their treatment plan and to keep an eye out for any negative effects, they had follow-up appointments every month.

Data collection and outcome measures

Prior to starting treatment, baseline data was gathered, such as demographics, mental diagnoses, and baseline sleep quality. Improvement in sleep quality, as measured by the PSQI at baseline, three months, and six months, was the main result. Evaluations of treatment safety and tolerability were among the secondary outcomes, which were tracked by documenting participant-reported adverse events during the study. Furthermore, daytime drowsiness was assessed using the Epworth drowsiness Scale (ESS), and overall therapy efficacy was measured using the Clinical Global Impressions-Improvement (CGI-I) scale.

Statistical analysis

IBM SPSS Statistics for Windows, Version 26.0 (Released 2019; IBM Corp., Armonk, New York, United States) was used to analyze the data. Demographic information, baseline sleep quality, and follow-up results for each of the three groups were compiled using descriptive statistics. Differences in primary and secondary outcomes across the therapy groups were evaluated using comparative methods, such as ANOVA and chi-square testing. To examine how each group's sleep quality changed over time, repeated measures ANOVA was used. For all analyses, a p-value of less than 0.05 was deemed statistically significant.

## Results

The study involved 175 individuals, with 58 treated with melatonin, 59 with trazodone, and 58 with doxepin. There was no discernible age difference between the groups, with the mean age being 39.2 years (SD = 11.4) (p = 0.71). The gender distribution did not differ significantly across the groups, with 98 (56%) of the participants being female (p = 0.82). All groups had severe sleep disruptions, as indicated by the average baseline PSQI score of 14.3 (SD = 3.1) (p = 0.59). The baseline characteristics showed no significant differences: for melatonin, 32 (55%) were female, with a mean PSQI score of 14.4 (SD = 3.0); for trazodone, 34 (58%) were female, with a mean PSQI score of 14.1 (SD = 3.3); and for doxepin, 32 (55%) were female, with a mean PSQI score of 14.3 (SD = 3.2). Additionally, the baseline ESS scores were similar across the groups, with melatonin having a mean score of 12.2 (SD = 4.1), trazodone having 12.0 (SD = 3.9), and doxepin having 12.4 (SD = 4.2), with no significant differences observed (p = 0.66) as shown in Table 1.

**Table 1:** Baseline Characteristics of Participants Continuous variables are expressed as mean ± SD, and categorical variables are presented as n (%), where n represents the frequency of participants and % denotes the percentage. Baseline characteristics were compared using ANOVA for continuous variables (age, PSQI, ESS) and chi-square tests for categorical variables (gender). P-values < 0.05 were considered statistically significant. PSQI: Pittsburgh Sleep Quality Index; ESS: Epworth Drowsiness Scale

Characteristic	Melatonin (n=58)	Trazodone (n=59)	Doxepin (n=58)	Test Statistic	p-value
Age	38.7 ± 11.1	39.5 ± 10.9	39.3 ± 12.2	F = 0.34	0.71
Female	32 (55%)	34 (58%)	32 (55%)	χ² = 0.39	0.82
Baseline PSQI score	14.4 ± 3.0	14.1 ± 3.3	14.3 ± 3.2	F = 0.53	0.59
Baseline ESS score	12.2 ± 4.1	12.0 ± 3.9	12.4 ± 4.2	F = 0.42	0.66

At baseline, three months, and six months, PSQI scores were evaluated. The mean PSQI reduction at three months was 4.2 points (SD = 1.9) for the melatonin group, 5.3 points (SD = 2.1) for the trazodone group, and 5.0 points (SD = 1.8) for the doxepin group. The mean decreases in PSQI scores by six months were 6.8 (SD = 2.1) for doxepin, 7.0 (SD = 1.9) for trazodone, and 6.1 (SD = 2.0) for melatonin. Although there were only slight differences between groups, a repeated measures ANOVA revealed a significant time effect (p < 0.001) and a group × time interaction (p = 0.03), suggesting that trazodone was linked to the most increase in sleep quality over time as shown in table 2.

**Table 2:** Change in PSQI scores over time Changes in PSQI scores over time were analyzed using repeated measures ANOVA. Asterisks indicate significance (*p < 0.05). PSQI: Pittsburgh Sleep Quality Index

Time Point	Melatonin (mean ± SD)	Trazodone (mean ± SD)	Doxepin (mean ± SD)	Test Statistic	p-value
Baseline	14.4 ± 3.0	14.1 ± 3.3	14.3 ± 3.2	F = 0.53	0.59
3 Months	10.2 ± 2.7	8.8 ± 2.9	9.3 ± 2.8	F = 9.32	< 0.001*
6 Months	8.3 ± 2.5	7.1 ± 2.7	7.5 ± 2.6	F = 7.85	< 0.001*

By the six-month mark, all groups had experienced a decrease in daytime sleepiness as determined by the ESS, with the trazodone group exhibiting the biggest decrease (mean decrease = 4.8, SD = 1.6), followed by doxepin (mean decrease = 4.2, SD = 1.9) and melatonin (mean decrease = 3.9, SD = 1.7). Significant group differences were shown by comparative analysis (p = 0.04).

With 45 (76%) respondents describing their sleep as "much improved" or "very much improved," the trazodone group showed the greatest overall improvement on the CGI-I scale, compared to 40 (69%) for the doxepin group and 35 (60%) for the melatonin group (p = 0.02), as shown in Table 3.

**Table 3:** Secondary outcomes, ESS and CGI-I scores ESS scores were analyzed using ANOVA, and CGI-I improvement was compared using chi-square tests. A p-value < 0.05 is considered statistically significant. Significant differences are marked with asterisks (*p < 0.05). ESS: Epworth Drowsiness Scale; CGI-I: Clinical Global Impression-Improvement

Outcome measure	Melatonin (n=58)	Trazodone (n=59)	Doxepin (n=58)	Test Statistic	p-value
ESS score change (mean ± SD)	-3.9 ± 1.7	-4.8 ± 1.6	-4.2 ± 1.9	F = 3.24	0.04*
CGI-I improvement (% much improved or very much improved)	60%	76%	69%	χ² = 7.89	0.02*

**Figure 1:**
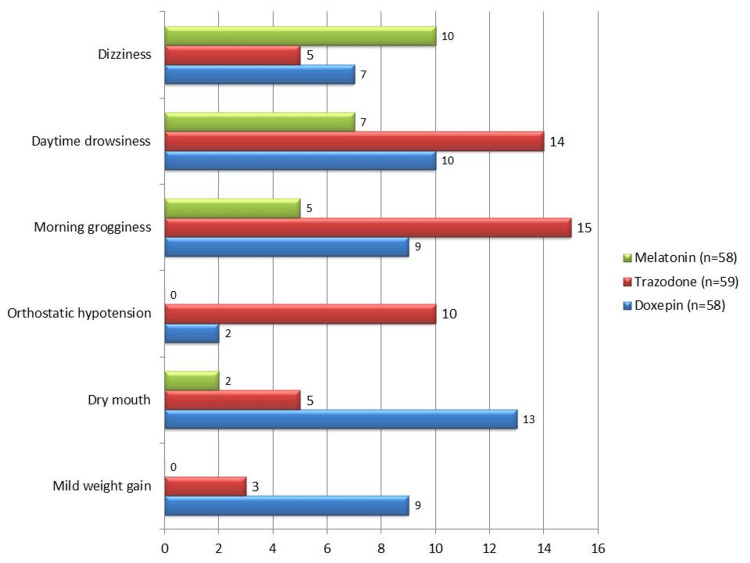
Adverse events seen in the different groups of patients Data is shown in percentage

The three therapy groups experienced the following adverse events: six (10%) patients on melatonin experienced dizziness, three (5%) patients on trazodone experienced it, and four (7%) patients on doxepin experienced it. There was no discernible difference between the groups (p=0.41). With no discernible difference (p=0.23), tiredness during the day was reported by four (7%) melatonin users, eight (14%) trazodone users, and six (10%) doxepin users. The trazodone group experienced morning grogginess more frequently (n=9, 15%) than the doxepin (n=5, 9%) and melatonin (n=3, 5%), and this difference was statistically significant (p=0.03). Six (10%) patients from the trazodone group, one (2%) from the doxepin group, and zero (0%) from the melatonin group experienced orthostatic hypotension; the differences between the groups were statistically significant (p=0.02). With a statistically significant difference (p=0.04), dry mouth was reported in one (2%) patient from the melatonin group, three (5%) from the trazodone group, and eight (13%) from the doxepin group. Lastly, a nearly significant difference (p=0.05) was seen in the reports of modest weight gain: two (3%) trazodone users, five (9%) doxepin users, and none of the melatonin users, as illustrated in Figure 1.

## Discussion

According to the study findings, doxepin and trazodone were the most successful treatments for enhancing sleep quality and decreasing daytime drowsiness. For patients who are worried about tolerability, melatonin is a good alternative because it was better tolerated and had fewer adverse effects, albeit being marginally less effective. Trazadone had the most negative effects, especially morning grogginess and orthostatic hypotension, although safety and tolerability profiles differed. These results offer a comparative analysis of the use of doxepin, trazodone, and melatonin in the treatment of sleep disturbances in mental health patients.

The findings of this study are in line with earlier investigations that demonstrate the differing levels of efficacy and tolerance of doxepin, trazodone, and melatonin in treating sleep problems in people with mental illnesses [16]. Previous research provides strong evidence for the higher effectiveness of trazodone in enhancing sleep quality and decreasing daytime drowsiness [17]. Our findings of significant PSQI reductions and higher CGI-I improvement in the trazodone group are consistent with the fact that trazodone dramatically improved sleep quality and decreased sleep latency in individuals with major depressive disorder [18]. Nonetheless, the trazodone group's higher prevalence of morning grogginess and orthostatic hypotension in the current study is consistent with earlier research showing that these side effects are frequently dose-dependent and more common in older adults [19]. The study highlights the necessity for personalized treatment strategies in psychiatric populations. Given the individual variability in responses to sleep medications, treatment plans must account for not only the effectiveness of a drug but also its safety profile and potential for adverse effects. Tailoring treatment options to the patient’s specific needs, including their age, comorbidities, and the nature of their psychiatric condition, will likely enhance both the safety and efficacy of sleep interventions.

Doxepin has been demonstrated to be efficacious with a comparatively superior side-effect profile, particularly at lower doses, and it also demonstrated notable increases in the quality of sleep [20]. Our results support earlier studies showing that low-dose doxepin mainly acts on histamine receptors, improving sleep duration while having fewer anticholinergic side effects than higher-dose tricyclic antidepressants. Melatonin demonstrated clinically significant improvements and a superior safety profile, despite being less effective than doxepin and trazodone at improving sleep quality [21]. This is in line with previous research that suggests melatonin's usefulness in regulating circadian rhythms rather than as a strong sedative [22]. This supports the idea that sleep therapies should be customized to meet the needs of each patient based on their effectiveness and tolerability, especially for psychiatric patients who might react sensitively to drugs [23].

Additionally, the study advances our knowledge of the ways in which these pharmaceuticals interact with psychiatric conditions. Although doxepin and trazodone provide significant benefits in the quality of sleep, they also carry the possibility of adverse effects, particularly in older people or those with cardiovascular conditions, which is consistent with previous worries raised in the literature [24]. Despite its relative lack of effectiveness in enhancing sleep quality, melatonin's safety profile highlights its use as a first-line treatment for individuals who are at a high risk of medication-related side effects [25]. The findings of this study emphasize the continued need to balance pharmacologic options with customized care plans that take into account both short-term benefits and long-term outcomes, such as medication tolerability and side effect burden, given the growing interest in non-pharmacological treatments for sleep disorders.

Strengths, limitations, and future directions

This study's prospective cohort design, which enabled the assessment of therapy efficacy and tolerability over a six-month period, was one of its main strengths. A research gap is also filled by the study's comparative strategy, which provides insightful information on the varying outcomes linked to doxepin, trazodone, and melatonin in a psychiatric setting. Nevertheless, the small sample size may restrict generalizability, and the use of self-reported measures such as PSQI may result in memory bias. Additionally, observer bias may result from the open-label approach when evaluating results. These results would be supported by bigger sample sizes and randomized, double-blind designs in future research, which would also help improve treatment guidelines for sleep disturbances in psychiatric patients. Furthermore, a more varied patient base including those with particular mental illnesses like schizophrenia, anxiety, and depression would help clarify how these drugs affect distinct patient populations.

## Conclusions

This study highlights the varying efficacy, safety, and tolerability of trazodone, doxepin, and melatonin in treating sleep disturbances in psychiatric patients. Trazodone was the most effective in improving sleep quality and reducing daytime drowsiness. However, it was associated with notable side effects such as morning grogginess and orthostatic hypotension. Doxepin also demonstrated strong efficacy, especially at lower doses, and had fewer adverse effects compared to trazodone. Melatonin, although less effective in improving sleep, was well-tolerated and a safer alternative for patients concerned with side effects. These findings emphasize the need for personalized treatment strategies to optimize outcomes and manage sleep disturbances in psychiatric populations.
